# Behavioral and technological interventions targeting glycemic control in a racially/ethnically diverse population: a randomized controlled trial

**DOI:** 10.1186/1471-2458-14-71

**Published:** 2014-01-23

**Authors:** Samuel N Forjuoh, Jane N Bolin, John C Huber Jr, Ann M Vuong, Omolola E Adepoju, Janet W Helduser, Dawn S Begaye, Anne Robertson, Darcy M Moudouni, Timethia J Bonner, Kenneth R McLeroy, Marcia G Ory

**Affiliations:** 1 Department of Family & Community Medicine, Scott & White Healthcare, College of Medicine, Texas A&M Health Science Center, Temple, TX, USA; 2 Department of Epidemiology & Biostatistics, School of Rural Public Health, Texas A&M Health Science Center, College Station, TX, USA; 3 Department of Health Promotion & Community Health Sciences, School of Rural Public Health, Texas A&M Health Science Center, College Station, TX, USA; 4 Department of Health Policy & Management, School of Rural Public Health, Texas A&M Health Science Center, College Station, TX, USA; 5 Department of Health and Kinesiology, Texas A&M University, College Station, TX, USA

**Keywords:** Chronic disease, Glycemic control, HbA1c, Self-management, Type 2 diabetes

## Abstract

**Background:**

Diabetes self-care by patients has been shown to assist in the reduction of disease severity and associated medical costs. We compared the effectiveness of two different diabetes self-care interventions on glycemic control in a racially/ethnically diverse population. We also explored whether reductions in glycated hemoglobin (HbA1c) will be more marked in minority persons.

**Methods:**

We conducted an open-label randomized controlled trial of 376 patients with type 2 diabetes aged ≥18 years and whose last measured HbA1c was ≥7.5% (≥58 mmol/mol). Participants were randomized to: 1) a Chronic Disease Self-Management Program (CDSMP; n = 101); 2) a diabetes self-care software on a personal digital assistant (PDA; n = 81); 3) a combination of interventions (CDSMP + PDA; n = 99); or 4) usual care (control; n = 95). Enrollment occurred January 2009-June 2011 at seven regional clinics of a university-affiliated multi-specialty group practice. The primary outcome was change in HbA1c from randomization to 12 months. Data were analyzed using a multilevel statistical model.

**Results:**

Average baseline HbA1c in the CDSMP, PDA, CDSMP + PDA, and control arms were 9.4%, 9.3%, 9.2%, and 9.2%, respectively. HbA1c reductions at 12 months for the groups averaged 1.1%, 0.7%, 1.1%, and 0.7%, respectively and did not differ significantly from baseline based on the model (*P* = .771). Besides the participants in the PDA group reporting eating more high-fat foods compared to their counterparts (*P* < .004), no other significant differences were observed in participants’ diabetes self-care activities. Exploratory sub-analysis did not reveal any marked reductions in HbA1c for minority persons but rather modest reductions for all racial/ethnic groups.

**Conclusions:**

Although behavioral and technological interventions can result in some modest improvements in glycemic control, these interventions did not fare significantly better than usual care in achieving glycemic control. More research is needed to understand how these interventions can be most effective in clinical practice. The reduction in HbA1c levels found in our control group that received usual care also suggests that good routine care in an integrated healthcare system can lead to better glycemic control.

**Trial registration:**

Clinicaltrials.gov Identifier: NCT01221090.

## Background

Diabetes education, particularly evidence-based approaches to diabetes self-care, is considered critical to achieving successful outcomes in diabetes management [1-3]. Several national organizations including the American Diabetes Association and the American Association of Diabetes Educators consider self-care an essential component of effective diabetes management [4-6]. Different approaches to improving glycemic control, the hallmark of diabetes treatment, have involved enhancing diabetes self-care processes using behavioral and technological programs. However, current literature provides mixed results on the effectiveness of self-care interventions in glycemic control and other related health measures. While most studies report positive short-term effects [7-9], others found no statistically significant differences in the longer term [10-12].

Behavioral interventions, such as the *Chronic Disease Self-Management Program* (CDSMP), offer the potential to improve overall health of individuals with diabetes, while preventing further decline in health status [13-15]. In a study evaluating changes in health behaviors, health status, and hospitalizations, CDSMP was found effective in improving health generally and resulted in lower hospitalization rates [13]. A recent meta-analysis of various self-care programs concluded a positive, but modest effect on numerous health-related outcomes [15]. However, until recently the CDSMP has not been delivered to substantial numbers of participants from racially/ethnically diverse backgrounds outside the original studies conducted by program developers [16].

Recent research also endorses the positive impact of technological interventions on diabetes management. As the use of portable blood glucose meters in the late 1980s began to change the way diabetes patients were monitored [17,18], researchers began experimenting with new technologies such as electronic diaries and the personal digital assistant (PDA) to assist with diabetes self-care in the early 2000s [19-21]. Diabetes self-care delivered via information technology is becoming an important factor in daily management for clinicians and patients [22]. These methods have been reported to assist patients to easily and accurately keep track of their self-care performance through immediate feedback [23]. In a pilot study, we tested the PDA’s feasibility in enhancing self-care activities of patients with type 2 diabetes mellitus (T2DM), identified which patients would benefit most from this technology, and assessed the effectiveness of our intervention on glycemic control [24]. The mean HbA1c decreased from 9.7% (83 mmol/mol) at baseline to 8.0% (64 mmol/mol) in 18 of 43 patients who completed the 6-month follow-up. The change in HbA1c was larger among regular and frequent PDA users [25]. This study did not, however, explore racial/ethnic differences or health disparities.

Despite concerted federal and state attempts to reduce health disparities over the past decades, national statistics still document substantial disparities in reported rates of diabetes for racial/ethnic minorities compared to non-minorities. For example, numerous studies document that African Americans and Hispanics experience higher rates of T2DM and associated cardiovascular diseases than other segments of the United States (U.S.) [26-29]. After adjusting for population age differences, 2007–2009 national survey data for people ≥20 years indicated that 7.1% of non-Hispanic whites, 8.4% of Asian Americans, 11.8% of Hispanics/Latinos, and 12.6% of African Americans had diagnosed diabetes [30]. Rates are also higher in rural and medically underserved populations due to the relative scarcity of healthcare providers, reduced access to needed healthcare, and less available electronic information systems [31-33]. As such, reducing and ultimately eliminating health disparities to achieve health equity for all groups continues to be a major public health objective [34]. While racial/ethnic minorities may still have reduced access to healthcare [35], their ownership rates of new technologies, such as mobile devices, may be at par with or even higher than their non-minority counterparts [36,37] and therefore benefit more from a technologically-assisted intervention such as use of a PDA.

The aim of this study was to test the effectiveness of two different diabetes self-care interventions on glycemic control in a large integrated healthcare organization in Central Texas that serves large racially/ethnically diverse populations. We also explored whether reductions in HbA1c will be more marked in minority persons with T2DM. This study is innovative in its comparison of behavioral and technological interventions as well as a combination of both interventions. Additionally, both clinical and behavioral outcomes were measured.

## Methods

### Design

This study was an open-label randomized controlled trial (RCT) designed to evaluate the effectiveness of two different T2DM self-care interventions (implemented singly and in combination) on glycemic control. Designed with the acknowledgment that both patients and researchers would be aware of the random assignment, the protocol consisted of screening potential subjects for eligibility, randomization to one of four study arms, and following them over a 24-month period. The primary end-point was change in glycated hemoglobin (HbA1c) from randomization/baseline to 12 months of follow-up. The study protocol was approved by the Institutional Review Boards (IRB) of Scott & White Healthcare System and Texas A&M Health Science Center. All qualified participants accepted the conditions of the study and gave informed written consent at enrollment/orientation. Enrollment occurred January 2009-June 2011 and data collection was completed in July 2012. We adhered to the CONSORT protocol [38].

### Setting, participants, and recruitment

Participants were recruited from seven participating clinics of a large integrated healthcare system, a university-affiliated, multi-specialty group practice associated with a 250,000-member Health Maintenance Organization in Central Texas. These clinics were selected based on their relatively higher numbers and overall percentage of African American and Hispanic patients diagnosed with T2DM. Potential participants were identified through electronic medical records if they: 1) had a diagnosis of T2DM; 2) were ≥18 years; 3) had a lab assessed HbA1c value ≥7.5% (≥58 mmol/mol) within the last six months; and 4) were able to communicate in English. Subjects were excluded if they: 1) had documented reports of alcoholism or drug abuse; 2) were pregnant or planning to become pregnant within 12 months; or 3) were unwilling to sign an informed consent. Recruitment was solicited by physicians within the seven clinics who agreed to invite their patients to participate in the study.

Physicians were provided with IRB approved invitation-to-participate letters and a list of their T2DM patients meeting the threshold HbA1c level at their last visit. Contact was initiated with potential subjects through physician-sent letters, describing the study and requesting a completed screening enrollment card if interested. Subjects who returned a screening enrollment card were contacted by project coordinators, who provided additional information and screened them to determine eligibility. To verify the inclusion and exclusion criteria, subject permission was obtained to review their medical records. Other recruitment strategies included oral referrals by physicians and patient educators and posting messages in waiting areas of study clinics.

Lab assessments were continuously monitored at each phase of recruitment to ensure enrolled participants had HbA1c values ≥7.5% (≥58 mmol/mol) within the last six months since individuals who previously met this criterion may no longer fulfill that requirement at orientation. A follow-up telephone interview was conducted to determine participation interest. Lab results were screened to ensure participant met qualifying HbA1c and if needed, tests were scheduled.

### Interventions

Consenting subjects were randomized to one of four arms: 1) CDSMP; 2) PDA; 3) a combination of both interventions (CDSMP + PDA); or 4) usual care (control). A fixed, equal allocation stratified randomization procedure was utilized, stratifying by clinic setting and race/ethnicity using Stata (version 9.2, 2006, StataCorp, LP, College Station, TX).

Participants randomized to the CDSMP arm were invited to attend a 6-week, classroom-based program for diabetes self-management. The effectiveness of CDSMP has been described elsewhere [13]. With the goal of increasing self-efficacy to ultimately decrease chronic disease related symptoms and avoidable healthcare utilization, CDSMP teaches participants techniques to facilitate enhanced decision making, action planning, and effective communication. CDSMP workshops were hosted in clinical environments and community-based settings. While fidelity to the individual classes was not monitored, CDSMP license requires that lay leaders use pre-scripted materials and that experienced master trainers/lay leaders (who underwent the four-day training program) lead the workshops.

Those randomized to the PDA arm were taught to use a diabetes self-care software, Diabetes Pilot™ (Digital Altitudes, Arlington Heights, IL), that was developed for the PalmOS® (Palm, Sunnyvale, CA) which was loaded on to the Tungsten™ E2 handheld device. The Diabetes Pilot allowed recording and some monitoring of blood glucose, blood pressure, medication usage, physical activity, and dietary intake on the PDA. One-on-one instruction by a project coordinator covering key areas such as data entry, food database utilization, and reports/graphing features was provided. Participants were instructed to enter information throughout the day and were encouraged to input daily. An instructional manual was provided and participants were asked to contact project coordinators with additional concerns/questions. Although proficiency with PDA use was not evaluated and individuals were provided additional guidance upon request, training effectiveness was not assessed.

Participants randomized to the CDSMP + PDA arm were given both interventions while those randomized to the control arm did not receive any treatment other than their usual clinical diabetes care, along with some publicly available Texas Diabetes Council patient education materials.

### Data collection

Study measures were obtained at orientation and at every six months over the 24 months of follow-up. Participants received monetary compensation in the form of a gift card for travel expenses and time, consisting of $20 at orientation and at the 12- and 24-month follow-up visits.

At orientation, a questionnaire was administered to obtain information on: 1) demographics including age, gender, and race/ethnicity; 2) technological experiences (e.g., any experience using computers, the internet, and a PDA); 3) self-reported health-related quality of life measures (e.g., number of days physical/mental health was not good); 4) diabetes self-care activities (number of days, 0–7, that any specific self-care activity was performed in the past week); 5) pain and fatigue measures (on a scale of 1–10, 1 indicating none and 10 severe); and 6) physical activity measures (e.g., number of physically active days in the past week). Questionnaires were administered every 6 months up to the 24-month follow-up visit. However, as our primary end point was 12-months, analyses were only conducted for this time period.

Anthropometric data, height, weight, and body mass index (BMI) and blood pressures were obtained at orientation and at subsequent follow-up visits. Participants who were unable to come in for their follow-up appointments had their height, weight, and blood pressure data abstracted from electronic health records (EHRs). Measures recorded fell within the range of 10 days prior to and 45 days after participants’ scheduled follow-up dates. This was done to obtain participant visits as close to their target dates as possible, but also allow for enough time after the target date to accommodate for scheduling errors (i.e., missed appointments, rescheduling).

Measures of HbA1c were collected from EHRs dating back six months prior to orientation to the last day of study participation. If a participant did not have any HbA1c value within the EHR for any particular follow-up visit, a lab test was scheduled to obtain a measure. Of the HbA1c collected six months prior to orientation, the value measured closest to the orientation date was considered as the baseline HbA1c value.

### Definition of a completed follow-up participation

A participant was considered to have completed a follow-up if there was an available HbA1c within the designated follow-up period, i.e., within the cut-off dates, defined as 45 days after the scheduled follow-up dates. For the 6-month follow-up measure, if at least one HbA1c was available after baseline and before the 6-month cut-off, the participant was considered to have completed a follow-up. For the 12-month follow-up measure, the designated range was between the 6-month cut-off date and the 12-month cut-off date. Participants who were unable to complete an assessment at one time period were not excluded from future assessments. For instance, if a participant did not have any HbA1c measured within the specified time period for their 6-month follow-up but had one available for their 12-month follow-up they were considered to have completed the 12-month follow-up, but not the 6-month.

### Outcome measures

The primary study outcome measure was change in HbA1c from randomization to 12 months of follow-up. Secondary outcome measures included BMI and blood pressure, along with several self-management behavioral measures (e.g., foot care) from randomization to 12 months of follow-up.

### Statistical analysis

Analysis was based on intent-to-treat. Descriptive statistics were used to describe baseline demographic, anthropometric, and clinical characteristics by study arm. Analysis of variance (ANOVA) was used to compare average changes in self-management behavior between study arms. To determine whether treatments had an effect on the rate of change in the level of HbA1c over time, we used a multilevel statistical model that included time as a continuous variable, measured in days, where 0 = baseline. The lowest level of the hierarchy (level 1) in this multilevel modeling was repeated measurements of HbA1c on each subject, with the participants themselves constituting the second level of the hierarchy (level 2). Forward selection was utilized, in which powers of time were added one at a time to the base model including treatment group effects only. Time and treatment effects were then added gradually and evaluated with likelihood ratio tests to assess any effect modification. HbA1c values included in the analysis were those falling within the time frame of six months prior to orientation until the 12-month follow-up cut-off point.

To explore whether health improvements following the interventions were more marked in racial/ethnic minority patients vs. non-Hispanic white populations, we ran another model that contained treatment by race/ethnicity interaction terms to test for differential impact of treatment by race/ethnicity. All analyses were conducted using Stata (version 12, 2012, StataCorp, LP, College Station, TX).

Sample size estimation was based on the two-level model for longitudinal change in HbA1c using the approximation formula by Fitzmaurice, et al [39]. The sample size estimation, based on 80% power and a two-sided significance level of .05, indicated that 75 participants per treatment group would be required to detect a statistically significant change in HbA1c of 0.5% for any pair-wise comparison of the treatment groups and the control group. Anticipating an attrition rate of 25%, we sought to enroll 100 patients in each arm so as to end up with the designated sample size. We also sought to achieve 50% minority (African American and Hispanics) and 50% non-minority participation within the study by over enrolling minority patients by an additional 30-40% so as to be able to conduct sub-analysis by race/ethnicity.

## Results

### Subject enrollment, participation, retention, and adherence

A total of 5,098 subjects were contacted by mail with the introductory, invitation-to-participate letter describing the purpose and details of the study. Of these, 3,201 were excluded based on their pre-screened lab results yielding HbA1c values <7.5% (<58 mmol/mol) or our inability to contact them. Of the remaining 1,897 potential subjects, 922 expressed an interest. However, 546 were ineligible based on HbA1c values <7.5% (<58 mmol/mol), lack of study interest, or inability to communicate in English, yielding 376 subjects who were subsequently randomized.

**Figure 1:**
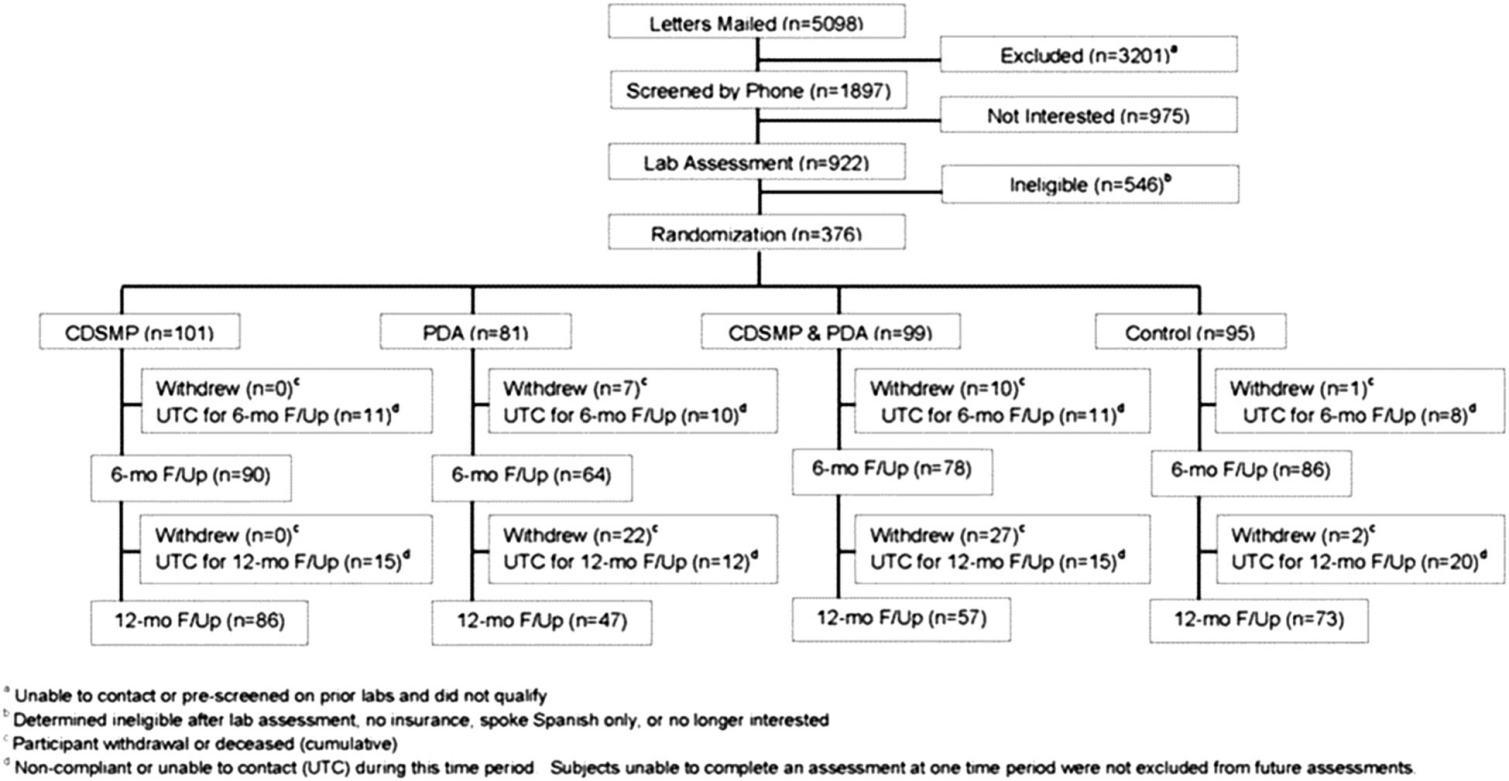
Consort table.

After 6 months of follow-up, 18 participants withdrew either on their own or due to death. Of this number, 7, 10, and 1 were from the PDA, CDSMP + PDA, and control group, respectively. The 6-month follow-up completion rates were 89%, 79%, 79%, and 91% for the CDSMP, PDA, CDSMP + PDA, and control group, respectively. After 12 months of follow-up, an additional 33 withdrew; 15 from the PDA group, 17 from the CDSMP + PDA group, and 1 from the controls, yielding 12-month follow-up completion rates of 85%, 64%, 64%, and 78% for the CDSMP, PDA, CDSMP + PDA, and control groups, respectively. The flow diagram of participant enrollment and disposition is summarized in a consort table in Figure 1.

Of the participants assigned to the CDSMP and CDSMP + PDA arms who started, 75.6% and 72.7%, respectively, attended 4 of 6 sessions required for successful completion. Participants assigned to the CDSMP + PDA and PDA arms made an average of 359 and 342 entries, respectively, on their PDAs over the one-year study period.

### Demographic data and baseline comparison of study population

Demographic and baseline clinical characteristics were generally comparable among the study groups (Table 1). The mean age of participants was 57.6 ± 10.9 years. Slightly more than a third (36.4%) was of minority status, self-reporting as either African American or Hispanic. The majority of participants received post-secondary education; 40% had attended some college or vocational school, 20% were college graduates, and 13% had completed higher forms of education. Approximately one-third reported annual incomes greater than $50,000, while almost 40% reported annual incomes between $25,000 and $49,999.

**Table 1:** Selected characteristics of study population at baseline

**Characteristics of participants**	**CDSMP**		**PDA**		**CDSMP + PDA**		**Controls**		**All**		
	**n = 101**		**n = 81**		**n = 99**		**n = 95**		**n = 376**		
Age (years), No. (%)											
18-34	4	(4.0)	1	(1.2)	2	(2.0)	1	(1.1)	8	(2.1)	
35-64	77	(76.2)	55	(67.9)	71	(71.7)	69	(72.6)	272	(72.3)	
65 or older	20	(19.8)	25	(30.9)	26	(26.3)	25	(26.3)	96	(25.5)	
Gender, No. (%)											
Female	54	(53.5)	47	(58.0)	53	(53.5)	53	(55.8)	207	(55.1)	
Male	47	(46.5)	34	(42.0)	46	(46.5)	42	(44.2)	169	(44.9)	
Minority^a^											
No	60	(59.4)	51	(63.0)	65	(65.7)	63	(66.3)	239	(63.6)	
Yes	41	(40.6)	30	(37.0)	34	(34.3)	32	(33.7)	137	(36.4)	
Hispanic, No. (%)											
No	81	(80.2)	62	(77.5)	76	(76.8)	80	(84.2)	299	(79.7)	
Yes	20	(19.8)	18	(22.5)	23	(23.2)	15	(15.8)	76	(20.3)	
Race-ethnicity, No. (%)											
Non-Hispanic white	58	(57.4)	49	(60.5)	61	(61.6)	58	(61.1)	226	(60.1)	
Non-Hispanic black	21	(20.8)	11	(13.6)	12	(12.1)	17	(17.9)	61	(16.2)	
Hispanic	20	(19.8)	19	(23.5)	22	(22.2)	15	(15.8)	76	(20.2)	
Other	2	(2.0)	2	(2.5)	4	(4.0)	5	(5.3)	13	(3.5)	
Education, No. (%)											
Less than high school	6	(5.9)	4	(4.9)	3	(3.0)	3	(3.2)	16	(4.3)	
Some high school	4	(4.0)	3	(3.7)	8	(8.1)	1	(1.1)	16	(4.3)	
High school graduate	16	(15.8)	20	(24.7)	17	(17.2)	21	(22.1)	74	(19.7)	
Some college/vocational school	46	(45.5)	34	(42.0)	33	(33.3)	36	(37.9)	149	(39.6)	
College graduate	16	(15.8)	13	(16.1)	24	(24.2)	21	(22.1)	74	(19.7)	
Graduate school	13	(12.9)	7	(8.6)	14	(14.1)	13	(13.7)	47	(12.5)	
Income, No. (%)											
< $15,000	12	(11.9)	11	(13.6)	7	(7.1)	9	(9.6)	39	(10.4)	
$15,000 - $24,999	11	(10.9)	14	(17.3)	19	(19.2)	16	(17.0)	60	(16.0)	
$25,000 - $49,999	41	(40.6)	37	(45.7)	32	(32.3)	30	(31.9)	140	(37.3)	
$50,000 - $75,000	12	(11.9)	12	(14.8)	23	(23.2)	17	(18.1)	64	(17.1)	
> $75,000	12	(11.9)	6	(7.4)	14	(14.1)	14	(14.9)	46	(12.3)	
Prefer not to answer	13	(12.9)	1	(1.2)	4	(4.0)	8	(8.5)	26	(6.9)	
BMI (kg/m^2^), No. (%)											
Normal	10	(10.3)	5	(6.3)	3	(3.2)	8	(8.7)	26	(7.1)	
Overweight	25	(25.8)	15	(18.8)	18	(19.0)	17	(18.5)	75	(20.6)	
Obese	62	(63.9)	60	(75.0)	74	(77.9)	67	(72.8)	263	(72.3)	
Age (years), Mean (±SD)	56.4	(±10.8)	57.7	(±10.8)	57.7	(±10.3)	58.5	(±11.9)	57.6	(±10.9)	
BMI (kg/m^2^), Mean (±SD)	33.5	(±8.0)	35.3	(±7.3)	34.6	(±6.3)	33.9	(±7.7)	34.3	(±7.4)	
SBP (mm/Hg), Mean (±SD)	131.9	(±14.1)	138.5	(±21.2)	136.2	(±19.1)	132.9	(±21.7)	134.8	(±19.3)	
DBP (mm/Hg), Mean (±SD)*	79.4	(±9.8)	73.6	(±11.0)	78.8	(±11.4)	75.8	(±13.6)	77.0	(±11.7)	
HbA1c (%), Mean (±SD)	9.4	(±1.7)	9.3	(±1.6)	9.2	(±1.4)	9.2	(±1.6)	9.3	(±1.6)	

^a^African American or Hispanic.

*Significant p < 0.05.

An overwhelming majority (92.9%) of the participants were either overweight or obese, with a mean BMI of 34.3 ± 7.4 kg/m [2]. While measures of systolic blood pressure were comparable among study arms, with a mean of 134.8 ± 19.3 mmHg, measures of diastolic blood pressure were significantly different (*P* < 0.002). The mean baseline HbA1c for participants was 9.3 ± 1.6% and did not differ significantly among the four groups.

### Changes in HbA1c from baseline to 12 Months

**Figure 2:**
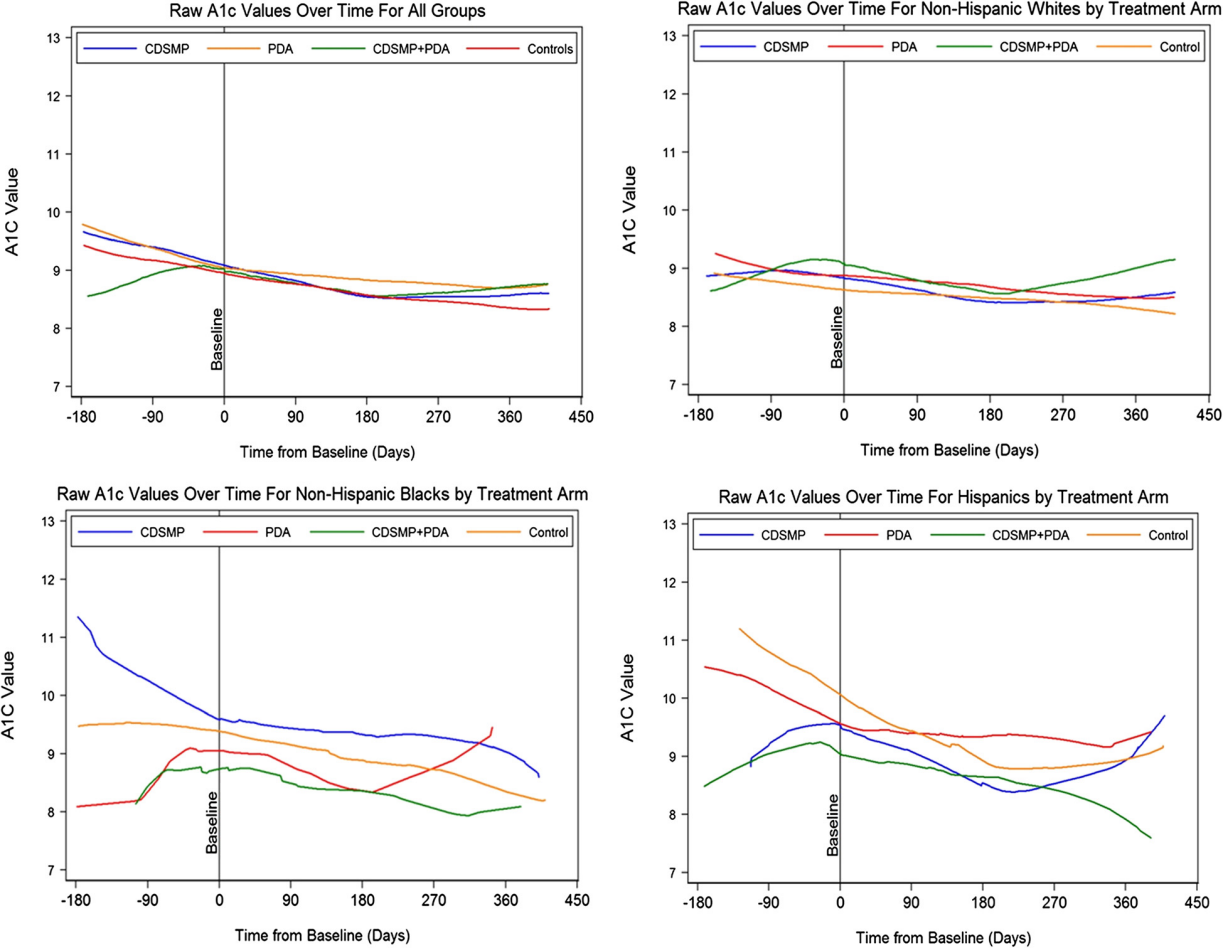
Lowess curves.

There were modest reductions in BMI and blood pressure from baseline to 12 months of follow-up for all four groups (table not shown). Similar results were observed with changes in HbA1c from baseline to 12 months of follow-up. Figure 2 displays *lowess* curves or trends of the raw data for HbA1c values for the four groups. The results of the multilevel statistical model are presented in Table 2. The reductions in HbA1c per day over the 12 months of follow-up for the control, CDSMP, PDA, and CDSMP + PDA groups were 0.002%, 0.003%, 0.002%, and 0.003%, respectively, which translated to HbA1c reductions of 0.7%, 1.1%, 0.7%, and 1.1%, respectively, from baseline to 12 months of follow-up. However, the main effect of treatment was not statistically significant (*P* = 0.771), implying no significant changes in HbA1c by treatment assignment. In addition, interactions with treatment-by-time and treatment-by-time squared did not reach statistical significance at *P* < 0.05.

**Table 2:** Model of HbA1c values of participants by treatment

**Model parameter**	**b(SE)**	**95% CI**	**Z**	***P*-value**
**Fixed effects**				
b_0_ (intercept)	9.029 (0.144)	8.746, 9.312	62.52	<0.001
b_1_ (Time)	-0.002 (0.001)	-0.004, -0.001	-3.13	0.002
b_2_ (Time^2^)	1.70 × 10^-6^ (2.27 × 10^-6^)	-2.75 × 10^-6^, 6.16 × 10^-6^	0.75	0.453
b_3_ (CDSMP)	0.114 (0.201)	-0.280, 0.508	0.57	0.571
b_4_ (PDA)	0.054 (0.214)	-0.366, 0.473	0.25	0.802
b_5_ (CDSMP + PDA)	-0.121 (0.204)	-0.520, 0.278	-0.60	0.551
b_6_ (CDSMP × Time)	-0.001 (0.001)	-0.003, 0.001	-1.17	0.242
b_7_ (PDA × Time)	7.22 × 10^-5^ (0.001)	-0.002, 0.002	0.06	0.950
b_8_ ((CDSMP + PDA) × Time)	-0.001 (0.001)	-0.003, 0.002	-0.61	0.543
b_9_ (CDSMP × Time^2^)	4.63 × 10^-6^ (3.16 × 10^-6^)	-1.55 × 10^-6^, 1.08 × 10^-5^	1.47	0.142
b_10_ (PDA × Time^2^)	2.04 × 10^-6^ (3.64 × 10^-6^)	-5.09 × 10^-6^, 9.17 × 10^-6^	0.56	0.575
b_11_ ((CDSMP + PDA) × Time^2^)	5.21 × 10^-6^ (3.47 × 10^-6^)	-1.59 × 10^-6^, 1.20 × 10^-5^	1.50	0.133
**Random effects**				
var(*u*_*1it*_ × *time*_*it*_)	0.002 (2.59 × 10^-4^)	0.002, 0.003	9.30	<0.001
var(*u*_*0i*_)	1.257 (0.056)	1.153, 1.372	22.53	<0.001
var(*e*_*it*_)	0.870 (0.024)	0.825, 0.918	36.39	<0.001

### Diabetes self-care activity monitoring during the intervention

The mean difference in the number of days (within the last 7 days), from baseline to 12 months of follow-up that participants reported using specific diabetes self-care activity features, measured by the Summary of Diabetes Self-Care Activities instrument, to assist with diabetes self-care activity monitoring were compared between treatment arms (table not shown). Participants in the PDA group reported eating more high-fat foods compared to their counterparts (*P* < .004). No other significant differences were observed.

### Exploratory sub-analysis by race/ethnicity

Health improvements following the introduction of self-management protocols were examined by race/ethnicity to explore whether there were any significant differences. *Lowess* curves for the raw data for HbA1c values by race/ethnicity are displayed in Figure 2. For this analysis, participants who self-reported as African American (n = 44), Hispanic (n = 61), or Caucasian (n = 168) were included in the model. Modest reductions occurred in HbA1c from baseline to 12 months of follow-up for all racial/ethnic groups (Table 3). However, the likelihood ratio test indicated that the respective treatments had a differential effect on the mean HbA1c by race/ethnicity at *P* < 0.05. On average, Hispanics who received the CDSMP + PDA intervention had a lower HbA1c compared to Caucasians who received the usual care. However, there was no statistically significant difference in HbA1c change over time by race/ethnicity. Additionally, Hispanics reported washing their feet significantly more than other racial/ethnic groups (*P* = 0.02) (table not shown).

**Table 3:** Model of HbA1c values of participants by race/ethnicity

**Model parameter**	**b(SE)**	**95% CI**	**Z**	***P*-value**
**Fixed effects**				
b_0_ (intercept)	8.765 (0.179)	8.414, 9.117	48.87	<0.001
b_1_ (Time)	-0.001 (0.001)	-0.003, 0.001	-1.04	0.297
b_2_ (Time^2^)	-1.21 × 10^-6^ (2.92 × 10^-6^)	-6.94 × 10^-6^, 4.52 × 10^-6^	-0.41	0.679
b_3_ (African American)	0.702 (0.374)	-0.031, 1.435	1.88	0.061
b_4_ (Hispanic)	1.242 (0.396)	0.466, 2.018	3.14	0.002
b_5_ (CDSMP)	0.099 (0.254)	-0.398, 0.597	0.39	0.696
b_6_ (PDA)	0.132 (0.266)	-0.391, 0.654	0.49	0.621
b_7_ (CDSMP + PDA)	0.177 (0.252)	-0.318, 0.671	0.70	0.483
b_8_ (CDSMP × African American)	0.247 (0.513)	-0.759, 1.253	0.48	0.631
b_9_ (CDSMP × Hispanic)	-0.699 (0.530)	-1.738, 0.340	-1.32	0.187
b_10_ (PDA × African American)	-0.620 (0.612)	-1.820, 0.579	-1.01	0.311
b_11_ (PDA × Hispanic)	-0.496 (0.542)	-1.559, 0.566	-0.92	0.360
b_12_ ((CDSMP + PDA) × African American)	-0.872 (0.575)	-1.999, 0.254	-1.52	0.129
b_13_ ((CDSMP + PDA) × Hispanic)	-1.226 (0.526)	-2.256, -0.195	-2.33	0.020
b_14_ (CDSMP × Time)	-0.002 (0.001)	-0.004, 0.001	-1.20	0.229
b_15_ (PDA × Time)	-1.62 × 10^-4^ (0.002)	-0.003, 0.003	-0.10	0.922
b_16_ ((CDSMP + PDA) × Time)	-0.002 (0.001)	-0.005, 0.001	-1.46	0.144
b_17_ (CDSMP × Time^2^)	5.64 × 10^-6^ (4.22 × 10^-6^)	-2.63 × 10^-6^, 1.39 × 10^-5^	1.34	0.181
b_18_ (PDA × Time^2^)	1.81 × 10^-6^ (4.86 × 10^-6^)	-7.71 × 10^-6^, 1.13 × 10^-5^	0.37	0.709
b_19_ ((CDSMP + PDA) × Time^2^)	8.85 × 10^-6^ (4.35 × 10^-6^)	3.19 × 10^-7^, 1.74 × 10^-5^	2.03	0.042
b_20_ (Control × African American × Time)	-0.001 (0.002)	-0.005, 0.002	-0.80	0.426
b_21_ (Control × Hispanic × Time)	-0.007 (0.002)	-0.011, -0.002	-2.86	0.004
b_22_ (CDSMP × African American × Time)	-0.001 (0.002)	-0.004, 0.003	-0.33	0.743
b_23_ (CDSMP × Hispanic × Time)	-0.003 (0.002)	-0.007, 0.001	-1.44	0.149
b_24_ (PDA × African American × Time)	0.001 (0.003)	-0.005, 0.007	0.21	0.837
b_25_ (PDA × Hispanic × Time)	-0.003 (0.002)	-0.007, 0.001	-1.61	0.108
b_26_ ((CDSMP + PDA) × African American × Time)	4.24 × 10^-4^ (0.003)	-0.006, 0.007	0.14	0.892
b_27_ ((CDSMP + PDA) × Hispanic × Time)	1.81 × 10^-4^ (0.002)	-0.004, 0.004	0.09	0.927
b_28_ (Control × African American × Time^2^)	2.68 × 10^-6^ (5.64 × 10^-6^)	-8.38 × 10^-6^, 1.37 × 10^-5^	0.47	0.635
b_29_ (Control × Hispanic × Time^2^)	1.57 × 10^-5^ (7.17 × 10^-6^)	1.68 × 10^-6^, 2.98 × 10^-5^	2.19	0.028
b_30_ (CDSMP × African American × Time^2^)	2.53 × 10^-6^ (5.48 × 10^-6^)	-8.21 × 10^-6^, 1.33 × 10^-5^	0.46	0.645
b_31_ (CDSMP × Hispanic × Time^2^)	5.54 × 10^-6^ (5.99 × 10^-6^)	-6.20 × 10^-6^, 1.73 × 10^-5^	0.93	0.355
b_32_ (PDA × African American × Time^2^)	2.53 × 10^-6^ (1.20 × 10^-5^)	-2.09 × 10^-5^, 2.60 × 10^-5^	0.21	0.832
b_33_ (PDA × Hispanic × Time^2^)	8.22 × 10^-6^ (6.56 × 10^-6^)	-4.64 × 10^-6^, 2.11 × 10^-5^	1.25	0.210
b_34_ ((CDSMP + PDA) × African American × Time^2^)	-3.82 × 10^-6^ (1.08 × 10^-5^)	-2.49 × 10^-5^, 1.73 × 10^-5^	0.36	0.722
b_35_ ((CDSMP + PDA) × Hispanic × Time^2^)	-2.75 × 10^-6^ (6.55 × 10^-6^)	-1.56 × 10^-5^, 1.01 × 10^-5^	-0.42	0.674
**Random effects**				
var(*u*_*1it*_ × *time*_*it*_)	0.002 (2.68 × 10^-4^)	0.002, 0.003	9.00	<0.001
var(*u*_*0i*_)	1.206 (0.056)	1.102, 1.320	21.68	<0.001
var(*e*_*it*_)	0.879 (0.025)	0.832, 0.929	35.66	<0.001

## Discussion

In this study, we sought to evaluate the individual and combined effects of two interventions – a behavioral and a technological intervention – targeting glycemic control via diabetes self-care. We found that participants receiving *any* of the treatments had similar rates of change in HbA1c over time compared to those who received usual care. This finding was somewhat surprising based on the results of our pilot study on PDA use to enhance diabetes self-care [25] and the fact that CDSMP has been found beneficial in lowering HbA1c among people with high levels [13,14,40]. In our pilot study, we found a 1.7% point reduction in HbA1c compared to the relatively small reductions found in this study (0.7%-1.1%). However, we also found a higher reduction in HbA1c at 6 months of follow-up for two of our treatment arms compared to 12 months of follow-up, supporting prior literature on the diminution of intervention effects over time [41]. In a meta-analysis of the effects of self-monitoring of blood glucose relative to usual care, an overall statistically significant decrease in HbA1c at 6 months of follow-up was reported. This effect, however, became non-significant at 12 months of follow-up [9].

Our results also corroborate the findings of another RCT group-based training for diabetes self-management, which reported no significant differences in HbA1c levels after 12 months of follow-up [10]. Nevertheless, our findings are less encouraging than others. A RCT reported a HbA1c reduction of 3.3% (13 mmol/mol) in the intervention arm at 6 months, and concluded that self-monitoring disease management strategy is able to improve metabolic control, primarily through lifestyle modifications leading to weight loss [8]. Another trial reported a significant decrease in HbA1c of 0.3% in the intervention group compared with the control group [42]. However, more than 30% were lost to follow-up. In our current study, it is important to note the improvement in the control group over time, suggesting a high level of routine diabetes care in an integrated healthcare system, which may involve close monitoring and self-care skill teaching as controls dropped 0.7% from baseline to 12 months.

Besides participants in the PDA group reporting a slightly higher intake of high-fat foods as was found in our pilot study [25], we found no other significant differences in our participants’ diabetes self-monitoring activity behaviors. There is a debate in the self-management field whether generic versus disease specific self-management is more beneficial [16,43]. While our view was that a generic program would be valuable for patients experiencing several comorbidities, more positive results might have been observed if the diabetes specific chronic disease self-management program was utilized (which was not evidence-based at the time of initial program selection for English speaking patients) [44].

Higher attrition among participants in the PDA and CDSMP + PDA groups deserves some comment. No doubt participant overburden with the extensive amount of data entry requirement may have resulted in this attrition. A similar attrition was observed in our pilot study [25] and several others [20,45]. Although we instituted several measures to mitigate this problem, such as providing intensive training via one-on-one instruction, several participants expressed frustration with the device, the program or both as their primary reason for discontinuing PDA use. The arrival of the revolutionary iPhone that coincided with our study may have also contributed to the higher attrition in study arms with the PDA [46]. Indeed, PDA usage, while being obsolete today, was the first foray in T2DM self-care via information technology. However, it may be more beneficial to shift our focus from diabetes self-management software designed for PDAs to more mainstream devices such as smartphones and tablets. These devices have already been accepted by the general public and integrating diabetes self-management programs on these platforms would yield a more seamless transition into an individual’s daily routine.

Results from our exploratory racial/ethnic sub-analyses were consistent with other studies in the literature. Contrary to our initial hypothesis, racial/ethnic differences in glycemic control did not diminish significantly over time, except for a slight reduction among Hispanics who received the CDSMP + PDA intervention. While we found no statistically significant racial/ethnic differences in HbA1c levels over time, Adams and colleagues noted persistent “white-black differences” in HbA1c levels among insured patients [47].

Our study had a few limitations that must be taken into consideration in the interpretation of the findings. There was differential dropout across interventions, suggesting that some intervention strategies are more difficult to learn and maintain than others. However, this initial analysis focused on an intent-to-treat analysis and means. Other future analyses will examine impacts among those who got recommended intervention dosages and examine different quartiles. It is possible that there could be a small but significant minority of participants who prove difficult to control and who thus mask improvements among others. We also recommend more careful monitoring of treatment fidelity to ascertain if behavioral and technological interventions were consistently delivered and enacted upon as recommended [48]. Due to the open label of our interventions, the behavior of participants in different arms of the study managed in the same clinics could have been affected by contact with the protocols in the different arms. Additionally, even though we tried to oversample minorities in order to obtain 50% minority and 50% non-minority participation, our final sample included only 34% minority participants. This prevented further analyses of race/ethnicity differences, allowing us to only provide information on racial/ethnic differences in an exploratory manner as the study was not powered to detect racial/ethnic differences. Finally, findings may not be completely generalizable to adults with uncontrolled T2DM since only 49% of eligible individuals screened by phone decided to participate in the study. Of those only 41% were randomized. Participants enrolled in our study may represent individuals who are more motivated or compliant compared to individuals with T2DM in the general population.

Aside from these limitations, some strengths of this study deserve mention. To our knowledge, it is first study to evaluate and compare the multilevel outcomes of behavioral and technological self-management techniques in a multi-setting population. It is also one of the first studies to evaluate and compare these interventions in a racially/ethnically diverse population in a practice setting outside of testing done by the original program developers. It therefore provides important exploratory data, shaping our knowledge and understanding of factors which may be important to minority and ethnic populations in adopting diabetes self-management techniques.

## Conclusions

In conclusion, we found that although behavioral and technological interventions can result in some modest improvements in glycemic control, these interventions did not fare significantly better than usual care in achieving glycemic control. More research is needed to understand how these interventions can be most effective in clinical practice. The reduction in HbA1c levels found in our control group that received usual care also suggests that good routine care in an integrated healthcare system can also lead to better glycemic control. We also recommend further studies that will be powered enough to examine racial/ethnic differences in glycemic control.

## Competing interests

None of the authors declare any competing financial interests.

## Authors’ contributions

SNF – Study conception, fund acquisition, supervision, data interpretation, manuscript preparation, and final review. JNB – Study conception, fund acquisition, supervision, data interpretation, manuscript preparation, and final review. JCH – Study conception, fund acquisition, data interpretation, manuscript preparation, and final review. AMV – Data analysis, data interpretation, manuscript preparation, and final review. OEA – Manuscript preparation, and final review. JWH – Data collection, manuscript preparation, and final review. DSB – Data collection, manuscript preparation, and final review. AR – Data collection, manuscript preparation, and final review. DMM – Data interpretation, manuscript preparation, and final review. TJB – Data interpretation, manuscript preparation, and final review. KRM – Fund acquisition, supervision, data interpretation, manuscript preparation, and final review. MGO – Study conception, fund acquisition, supervision, data interpretation, manuscript preparation, and final review. All authors read and approved the final manuscripts.

## Authors’ information

SNF – Professor and Director of Research.

## Pre-publication history

The pre-publication history for this paper can be accessed here:

http://www.biomedcentral.com/1471-2458/14/71/prepub
